# Tigecycline combined with bronchoscopic interventions in the treatment of macrolide-unresponsive *Mycoplasma penumoniae* pneumonia: A case report

**DOI:** 10.1016/j.heliyon.2024.e40058

**Published:** 2024-11-01

**Authors:** Xinyue Ma, Lei Tian, Shuyun Xu, Jin Shang

**Affiliations:** Tongji Hospital, Tongji Medical College, Huazhong University of Science and Technology, Wuhan, Hubei Province, 430030, China

**Keywords:** *Mycoplasma penumoniae* pneumonia, Macrolide-unresponsive, Tigecycline, Bronchoscopic interventions, Case report

## Abstract

*Mycoplasma penumoniae* (MP) is a common etiological agent of community-acquired pneumonia. However, there has been an increasing incidence of macrolide-unresponsive *Mycoplasma penumoniae* pneumonia (MUMPP) in recent years. The treatment of MUMPP requires further investigation. In this report, we describe a case of MUMPP complicated by secondary spontaneous pneumothorax. The patient was unresponsive to initial macrolide treatment and his pneumonia worsened with increasing hypoxemic respiratory failure. However, after receiving a novel tetracycline and a carbapenem antibiotic as anti-infective agents, glucocorticoid for anti-inflammatory and bronchoscopic interventions to clear the bronchial casts, his fever and hypoxia resolved, and his lung lesions had significantly improved. Symptomatic supportive measures, including supplemental oxygen, was provided for the management of spontaneous pneumothorax developed twenty days after discharge. At follow-up, he did not experience any more initial symptoms. All indexes remained normal for half a year. This study represents the initial investigation into the utilization of tigecycline in conjunction with bronchoscopic interventions including bronchoalveolar lavage (BAL) and bronchoscopic cryotherapy (BC) in the treatment of MUMPP, glucocorticoid can be considered for anti-inflammatory purposes, especially for patients with severe pneumonia. The findings from this case offer valuable insights into a potential therapeutic approach for individuals afflicted with MUMPP.

## 1 Introduction

*Mycoplasma penumoniae* (MP) is a prevalent causative agent of community-acquired pneumonia. The primary clinical symptoms observed in patients include fever and cough, with the majority of cases presenting as mild and non-specific [1]. Severe cases may manifest with both intrapulmonary and extrapulmonary complications, such as acute respiratory distress syndrome, myocarditis, and meningitis, occurring in 0.5%–2% of cases [2]. Macrolide antibiotics are presently the primary therapeutic option for MP pneumonia (MPP). However, there has been an increasing incidence of macrolide-unresponsive MPP (MUMPP), potentially linked to the widespread application of macrolide antibiotics [3], with reported drug resistance rates as elevated as 90 % in China [4]. Tigecycline, a novel tetracycline derivative, represents a promising alternative for anti-infective treatment in MPP [2].

What is more, bronchoscopic interventions including bronchoalveolar lavage (BAL) and bronchoscopic cryotherapy (BC) can significantly contribute to the etiological diagnosis and treatment of refractory pneumonia [5]. BAL not only facilitates the aspiration of airway secretions to reduce necrotic material and inflammatory mediators but also aids in the identification of pathogenic microorganisms through BALF. BC, a procedure involving the removal of bronchial tube obstructions by freezing with a cryoprobe, can effectively alleviate bronchial blockages and improve atelectasis [6,7].

However, limited cases investigating its application have been documented. In this case report, we describe a juvenile patient with severe MPP who exhibited unresponsiveness to macrolide therapy. The patient's condition significantly improved after receiving the novel tetracycline, a carbapenem antibiotic, glucocorticoid, and bronchoscopic interventions.

## 2 Case presentation

A 17-year-old male patient, was admitted to Tongji Hospital, Tongji Medical college, Huazhong University of Science and Technology presenting with a persistent fever and cough for one week, whose maximum body temperature reached 40.2 °C. The patient also reported experiencing headache, weakness, and severe paroxysmal cough producing yellow sputum. Prior to admission, he underwent treatment at a local hospital where a respiratory tract nucleic acid test confirmed MP infection, and chest computed tomography (CT) displayed patchy consolidation in the lower lobe of the right lung five days earlier. Despite three days of treatment with azithromycin and piperacillin tazobactam sodium, the patient's fever persisted, reaching a peak body temperature of 40.2 °C at the local hospital. Subsequently, the physician transitioned to meropenem; however, the patient continued to exhibit intermittent fever, registering a temperature of 40.0 °C.

The physical examination results were as follows: the patient exhibited a body temperature of 39.8 °C, a pulse rate of 151 beats per minute, a respiration rate of 22 breaths per minute, and a blood pressure reading of 75/52 mmHg (1 mmHg = 0.133 kPa). Upon arrival at the ward, the patient ambulated independently and was alert. Absence of cyanosis in the oral cavity or lips, jaundice of the skin or sclera, rash, ecchymoses, or palpable superficial lymph nodes was noted. Coarse breath sounds were auscultated bilaterally, accompanied by dry rales. No other significant abnormalities were noted.

**Fig. 1:**
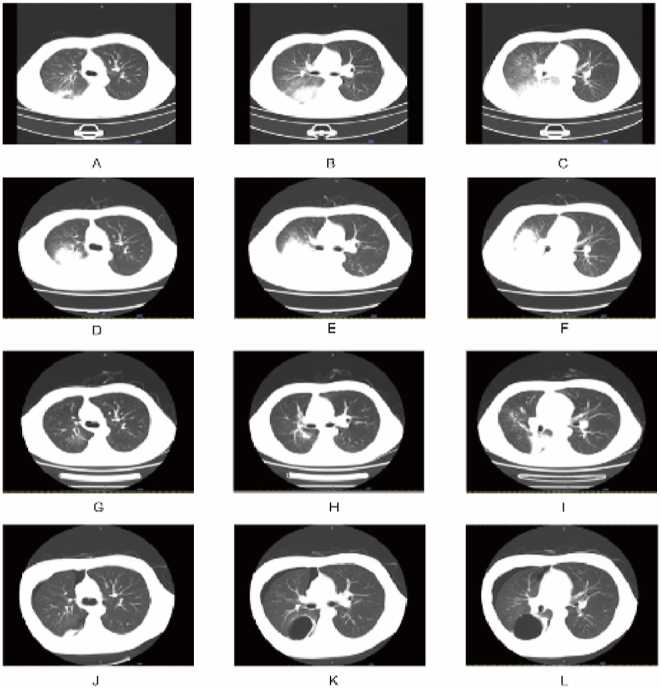
Chest computed tomography (CT) images of an adolescent patient with macrolide-unresponsive *Mycoplasma penumoniae* pneumonia. (A–C) Chest CT on hospital Day 1, showing that patchy consolidation of the right lung, significant in the right lower lung. (D–F) Chest CT on hospital Day 9, showing that the original consolidation in the right lung had become enlarged and new patchy consolidations had appeared in the bilateral lower lobes. (G–I) Chest CT on hospital Day 17, showing significant resolution of the bilateral lung lesions. (J–L) Chest CT on the 2nd day of readmission, showing a right pneumothorax with intrapulmonary bullae.

The chest CT showed patchy consolidation in the right lung, predominantly affecting the lower right lung (Fig. 1). Laboratory findings indicated the following: Routine blood work showed a white blood cell (WBC) count of 10.13 ∗10^9^/L (normal range: 4.1–11.0 ∗10^9^/L), a neutrophil count of 8.90 ∗10^9^/L (normal range: 1.8–8.3 ∗10^9^/L), and a lymphocyte count of 1.01 ∗10^9^/L (normal range: 1.2–3.8 ∗10^9^/L). The alanine aminotransferase (ALT) was 39 (normal range, ≤41) U/L, the aspartate aminotransferase (AST) was 57 U/L (normal range: ≤40 U/L), the lactate dehydrogenase (LDH) was 511 U/L (normal range: 135–225 U/L), the serum creatinine (Scr) concentration was 92 μmol/L (normal range: 59–104 μmol/L) (Table 1). The patient underwent the nucleic acid amplification of SARS-CoV2 by PCR, yielding a negative result. The ELISA test for immunoglobulin M (IgM) antibodies against MP and *Chlamydia pneumoniae* were negative, but the test for immunoglobulin G (Ig G) antibodies against MP returned positive. The patient's oxygenation index was measured at 265. On the third day of admission, bronchoalveolar lavage (BAL) was performed. Bacterial, fungal, and tuberculosis cultures from the bronchoalveolar lavage fluid (BALF) were all negative. Through comparison of the obtained sequences in the National Center for Biotechnology Information (NCBI), MP was detected by metagenomic next-generation sequencing (mNGS). Thus, the patient was diagnosed with MPP complicated with typeⅠrespiratory failure.

**Table 1:** Clinical laboratory results.

Measure	Normal range	The 1st day of admission	The 5th day of admission	The 7th day of admission	The 9th day of admission	The 15th day of admission	The 18th day of admission
Routine bloodwork							
WBC ( × 10^9^/L)	4.1–11.0	10.13	6.92	5.93	8.55	7.85	6.93
Neutrophil ( × 10^9^/L)	1.8–8.3	8.90	5.69	4.64	6.45	4.97	4.09
Lymphocyte ( × 10^9^/L)	1.2–3.8	1.01	0.83	0.70	1.07	1.75	1.95
Inflammatory index							
Procalcitonin (ng/mL)	0.02–0.05	–	1.25	0.32	0.21	–	–
Biochemical indexes							
ALT (U/L)	≤41	39	111	118	100	17	17
AST (U/L)	≤40	57	159	136	71	19	20
LDH (U/L)	135–225	511	728	527	527	347	287
Scr (umol/L)	59–104	92	56	53	56	54	72

WBC, white blood cell; ALT, alanine aminotransferase; AST, aspartate aminotransferase; LDH, lactate dehydrogenase; Scr, serum creatinine.

**Fig. 2:**
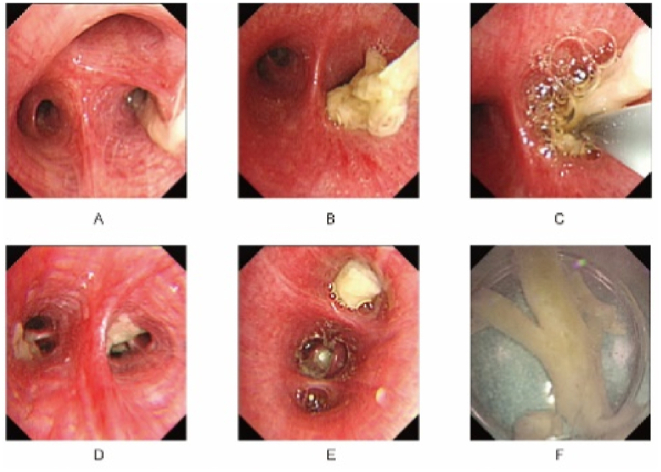
Bronchoscopy results for a patient with macrolide-unresponsive *Mycoplasma penumoniae* pneumonia. (A–F) Bronchoscopy shows a large number of sputum bolt obstructing lumen.

Upon admission, the patient commenced a daily dose of 0.4g moxifloxacin, and non-invasive ventilation was initiated to aid ventilation owing to a clinical diagnosis of pneumonia complicated by type Ⅰ respiratory failure. Nonetheless, the patient's fever persisted, escalating to 40 °C. Furthermore, respiratory failure symptoms deteriorated, leading to a decrease in the oxygenation index to 86.3. Bedside bronchoscopy revealed a a significant accumulation of sputum clots obstructing the lumen in the posterior segment of the right upper lobe bronchus, as well as the right middle segment, and the right middle and lower lobe bronchi. Regrettably, the removal of these clots with acetylcysteine proved ineffective. Subsequently, these clinical observations led to the confirmation of a severe and life-threatening case of Mycoplasma pneumoniae pneumonia. Consequently, the patient's anti-infective drug was changed to tigecycline (a dose of 50 mg by intravenous infusion every 12 hours) and treatment with methylprednisolone sodium succinate (MSS) was initiated at a daily dose of 40mg from the 6th day of admission. Moreover, imipenem and cilastatin sodium were administered at a dosage of 1.0 g via intravenous infusion every 8 hours, taking into account the potential involvement of secondary pathogen infections in the deterioration of the patient's condition. Laboratory tests conducted on the 7th day of admission, revealed a positive IgM antibody for MP with a value of 1.64 S/CO. As a result, the dose of MSS was reduced to 20mg intravenously once a day on the 9th day of admission, due to significant improvement in the patient's laboratory indexes (Table 1). Subsequent BALF tests not only confirmed the presence of *Acinetobacter junii* by culture, but also detected the presence of MP DNA by PCR. Following these developments, the patient's temperature normalized, and he was successfully weaned off the noninvasive ventilator after the 12th day of admission. The intravenous MSS medication was then switched to oral prednisone acetate tablets at a dosage of 15mg every 24 hours starting from the 15th day of admission. Bronchoscopy was performed on the 3rd day, the 6th day, the 9th day and the 16th day of admission to clear the bronchial casts (Fig. 2). The patient's symptoms showed significant improvement and the chest CT scan indicated gradual shrinkage of the lesions (Fig. 1). Consequently, the patient was discharged on the 19th day, with a recommendation to continue using oral minocycline for one more week.

**Fig. 3:**
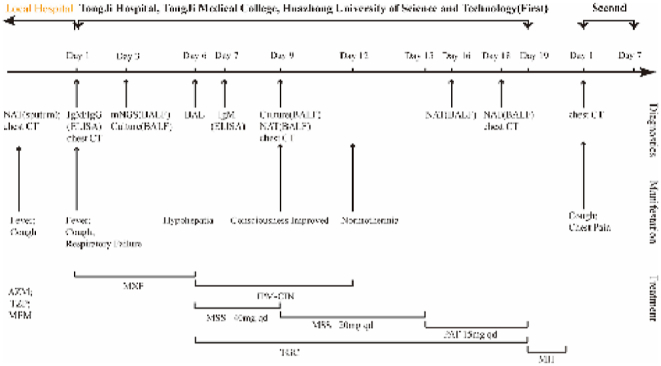
Timeline of the patient's diagnosis, manifestation and treatment.

Twenty days after discharge, the patient had chest pains after laughing out loud and was readmitted due to spontaneous pneumothorax. The chest CT showed a right pneumothorax with intrapulmonary bullae (Fig. 1). No DNA or RNA of MP was detected by PCR. Symptomatic supportive measures, including supplemental oxygen, was provided and the patient was discharged on the 7th day after showing improvement (Fig. 3). And the patient is currently healthy at school now.

## 3 Discussion

The clinical and imaging manifestations of patients with MPP often lack specificity, underscoring the importance of promptly identifying the pathogen for appropriate drug selection. BALF, a lower respiratory tract specimen, offers advantages by minimizing the influence of upper airway colonization. Notably, BALF demonstrates higher diagnostic efficiency compared to sputum and blood. The sensitivity and specificity of BALF range from 40 % to 93 % (median 73 %) and 45 %–100 % (median 82 %), respectively [8], providing valuable guidance for drug therapy [5].

In general, MPP is a self-limited condition with low fatality rates. However, approximately 0.5 % of MPP cases can progress to severe forms, which may present with intrapulmonary complications like plastic bronchitis (PB) and pulmonary embolism, as well as extrapulmonary complications such as encephalitis and myocarditis [9]. Complications like lung bullae, emphysema and spontaneous pneumothorax are rare in MPP [10]. The development of these complications may be attributed to direct cell damage, inflammatory injury induced by cytokines, and immune evasion subsequent to MP infection [11]. Co-infection with other pathogens can increase the chance of extrapulmonary complications, aggravate MPP, and even lead to death. The co-infection rate of MP and other pathogens ranged from 10 to 56.1 % [12].

The primary emphasis of therapy should center on promptly recognizing and managing severe and fulminant MPP. It is advisable to commence treatment within 5–10 days following the onset of fever. Essential strategies encompass offering symptomatic relief and supportive care, administering suitable anti-infective agents, employing glucocorticoids as warranted, and contemplating bronchoscopic interventions [13].

Macrolides are the preferred antibiotics for treating MP in children [9]. However, there has been a noticeable increase in the incidence of MUMPP in recent years, particularly among children [3]. MP is a self-replicating prokaryotic microorganism, which lacks a cell wall and exhibits slow growth, with a highly stable genome. Macrolides inhibit protein synthesis by binding to domains II and/or V of the 23S rRNA in the 50S bacterial ribosomal subunit. MUMPP, characterized by the lack of clinical response to macrolide treatment, may be related to mutations in domain V of 23S rRNA, with the most common mutation being A-G at position 2063 (A2063G), followed by A2064G and A2063C [4].

In this report, we described a severe life-threatening case of MPP that was unresponsive to initial macrolide treatment but exhibited improvement after treatment with tigecycline. Unfortunately, our detection did not contain 23S ribosomal RNA (rRNA) mutations that commonly associate with macrolide resistance. Therefore, we could only consider it as the MUMPP, and could not determine whether it is the macrolide-resistant MPP (MRMPP). Considering that the clinical deterioration observed in the patient might be attributed to excessive immune inflammatory responses, intrapulmonary complications and co-infection with other pathogens, we added a carbapenem antibiotic for anti-infective treatment, glucocorticoid with immunomodulatory effects and repeated bronchoscopy for cast removal. Subsequent BALF tests confirmed the presence of MP and *Acinetobacter junii*. Fortunately, *Acinetobacter junii* is susceptible to carbapenem antibiotics and tigecycline [14].

Fluoroquinolones have been linked to potential harm to the articular and musculoskeletal systems [15], and are recommended for use only in patients aged 18 years and older. Tetracyclines have been associated with negative effects on bone development, such as tooth enamel hypoplasia and permanent tooth discoloration in young children [15]. Therefore, these medications should be exclusively prescribed to children aged 8 and above. Minocycline and doxycycline are recommended for the treatment of MUMPP [13]. Tigecycline, a derivative of minocycline, has broad spectrum activity against various pathogenic microorganisms, especially MP and resulted in rapid resolution of clinical manifestations in adults with MUMPP [16]. Furthermore, lefamulin [17] and omadacycline [18] have recently gained significant attention.

Excessive secretion of airway mucus caused by MP can lead to local obstruction of the bronchial lumen, potentially resulting in pulmonary consolidation and atelectasis. Two strategies can be employed to address this issue. Firstly, bronchoscopy can be used to directly aspirate airway secretions and perform BAL to eliminate necrotic material and alleviate inflammation. Secondly, bronchoscopy can be employed to clear obstructions by employing biopsy forceps directly or through BC, effectively unblocking the bronchus and improving atelectasis. BC involves using a cryoprobe to freeze and remove obstructions within the bronchial tube, typically inserted through a flexible bronchoscope. Presently, bronchoscopic techniques are extensively applied for the diagnosis and management of pulmonary conditions. They facilitate direct visualization of airway and mucosal abnormalities, pathogen identification through BALF, and reduction of inflammatory mediators, potentially reducing the need for antibiotics and enhancing of ventilation function [6,7].

The infection of MP involves cellular immunity and humoral immunity. Elevated levels of inflammatory cytokines have been observed in patients with MUMPP compared to those with macrolide-sensitive MP infection [19]. Glucocorticoid can rapidly regulate the phagocytic and release capacities of inflammatory mediators, exerting potent anti-inflammatory and immunomodulatory effects while gradually enhancing microcirculation. Therefore, in cases where patients with prolonged fever due to heightened host immune responses fail to show temperature reduction despite antibiotic treatment, glucocorticoid therapy may be considered as an alternative approach to attenuate excessive host immune responses [20]. What's more, glucocorticoid can reduce the risk of MV utilization and the duration of ICU and hospital in the treatment of patients with severe pneumonia [21]. Various guidelines are available delineating the optimal timing and dosing regimens for corticosteroid therapy [9].

## 4 Conclusion

We report a case of MPP that was unresponsive to macrolide therapy and resulted in secondary spontaneous pneumothorax. The patient's condition notably improved following treatment with a novel tetracycline regimen in conjunction with the glucocorticoid and bronchoscopic interventions. Symptomatic supportive measures, including supplemental oxygen, were administered for the management of spontaneous pneumothorax. This case underscores the significance of appropriate antibiotic selection and bronchoscopic interventions in MUMPP. Glucocorticoids may be considered for their anti-inflammatory properties in cases of severe pneumonia. The case study aims to offer insights into a potential treatment approach for patients with MUMPP.

## CRediT authorship contribution statement

**Xinyue Ma:** Writing – original draft, Project administration, Methodology, Investigation, Formal analysis, Data curation, Conceptualization. **Lei Tian:** Writing – review & editing, Data curation. **Shuyun Xu:** Writing – review & editing, Formal analysis, Data curation. **Jin Shang:** Writing – review & editing, Supervision, Software, Funding acquisition, Data curation.

## Ethics statement

The study was conducted in accordance with the Declaration of Helsinki, and approved by the Ethics Committee of Tongji Hospital, Tongji Medical College, Huazhong University of Science and Technology (TJ-IRB20230515). Written informed consent has been obtained from the patient and the guardians to publish this paper.

## Data and code availability statement

Data included in article/supplementary material is referenced in the article.

## Declaration of competing interest

The authors declare the following financial interests/personal relationships which may be considered as potential competing interests: Jin Shang reports financial support was provided by the Program of The 10.13039/501100001809National Natural Science Foundation of China (No. 81900092). If there are other authors, they declare that they have no known competing financial interests or personal relationships that could have appeared to influence the work reported in this paper.
